# A Successful Esthetic Approach of Gingival Depigmentation Using Microneedling Technique and Ascorbic Acid (Vitamin C)

**DOI:** 10.1155/2022/3655543

**Published:** 2022-04-25

**Authors:** Diana Mostafa, Shaden M. Alotaibi

**Affiliations:** ^1^Clinical Periodontology Department, Faculty of Dentistry, Alexandria University, Alexandria, Egypt; ^2^Preventive Dental Sciences, Vision colleges for dentistry and Nursing, Riyadh, Saudi Arabia

## Abstract

A gingival depigmentation is a periodontal plastic procedure that is performed in order to remove melanocytic pigmentation. A variety of different modalities have been proposed for removing hyperpigmentation involving surgical scraping, gingival autograft, cryotherapy, electrosurgery, and lasers. However, the microneedling technique is a nonsurgical procedure that creates microholes to facilitate the penetration of topical medications across the connective tissues. *Case Description*. A healthy female patient aged 25 years with a pigmented gingiva seeking gingival depigmentation. On examination, a dark brown ribbon of hyperpigmentation was observed within the mandibular attached gingiva. The case was diagnosed as physiological moderate gingival pigmentation (pigmentation index score = 3). The patient was interested in achieving aesthetic results with minimally invasive, nonexpensive procedures. Based on the patient's concerns, the microneedling technique using vitamin C was suggested and consented. We used a dermapen device to microneedle the gingiva until bleeding pinpoints were observed; then, topical ascorbic acid was applied. After 3 days, our outcomes revealed an excellent aesthetic pink gingival appearance. *Conclusions and Practical Implications*. Compared to other minimally invasive techniques, our technique is less expensive and more risk-free. Our novel technique of using dermapen and topical ascorbic acid has shown promising results to our case which gives new perspectives for its application in gingival depigmentation.

## 1. Introduction

Since perioaesthetics have become a major demand in dentistry, treatment protocols should not only address functional and biological problems but also establish harmony between white and pink (teeth and gingiva). A typical macroanatomical feature of healthy gingiva is its pink colour which diverges into various shades depending on the degree of keratinization, the thickness of the gingiva, the degree of vascularization, reduction of haemoglobin, and the presence of melanocytic cells [1].

Hyperpigmented gingiva is caused by a wide range of external or internal influences. It is maybe due to physiological or pathological factors, which cause the excessive deposition of melanin granules in melanocytes in the basal and suprabasal layers of the epithelium [2]. Gingival hyperpigmentation can cause a cosmetic problem that may have an adverse psychological impact on patients, especially those with short lips and high smile lines. However, gingival pigmentation (GP) tends to be more prominent among dark-skinned individuals. It is more likely to be observed in the anterior area than the posterior, and the mandible is more commonly affected than the maxilla. The degree of GP is affected by melanocyte activity, melanosomes number, dispersion degree, and degradation of the pigment [1, 2].

Gingival hyperpigmentation was classified according to melanin index by Hanioka et al. [3] into three classes; class 0 indicates no pigmentation, class 1 represents solitary units of pigmentation in papillae only, and class 2 displays a continuous ribbon of gingival pigmentation (GP). In addition, the oral pigmentation index was introduced by Dummett and Gupta in 1971 [4] as scores for GP according to its colour degree; score 1 is given to pink gingiva (no pigmentation), score 2 indicates light brown pigmentation (mild pigmentation), score 3 represents medium brown or mixed brown pink and brown pigmentation (moderate pigmentation), and score 4 indicates deep brown or bluish-black pigmentation (heavy pigmentation). In 2017, the American Academy of Periodontology and the European Federation of Periodontology proposed a new classification of periodontal and peri-implant diseases [5] in which gingival colour alterations are diagnosed as “gingival pigmentations” and it was classified into melanoplakia, smoker's melanosis, drug-induced pigmentations, and amalgam tattoo.

Gingival depigmentation (GD) is considered a periodontal plastic procedure whereby melanocytic pigmentation is removed. A variety of different modalities have been proposed for removing hyperpigmentation involving bur abrasion, surgical scraping, gingival autograft, cryotherapy, electrosurgery, and laser [6]. In addition, some studies suggest that ascorbic acid (vitamin C) could be used to treat gingival pigmentation [7].

However, the microneedling (MN) technique is a nonsurgical procedure that is known as collagen induction therapy involving repetitive punctures on the skin. In dermatology, MN has been utilized considerably in recent years as it is an effective, simple, economical, well-tolerated, and cosmetically and therapeutically beneficial procedure [8]. The MN serves to separate the cells instead of cutting through forming microconduits which increases the skin's permeability and blood flow into the epidermis. This process facilitates the penetration of topical medications across the stratum corneum layer. Besides, growth factors are produced promoting the regeneration of collagen and elastin [8, 9].

However, ascorbic acid (AA) has been demonstrated as a water-soluble antioxidant and an essential nutrient for collagen biosynthesis [10]. It plays a role in immunomodulation as well as the elimination of the hyperpigmented spots [11] by interacting with the copper ions at the tyrosinase active site and inhibiting the activity of the enzyme tyrosinase, thereby diminishing melanin production [10].

While many clinical investigations have been documented, on the effectiveness of the MN technique in treating scars and wrinkles, promoting skin rejuvenation, and managing pigmentation disorders [8, 12–14], scarce dental studies have been conducted on its application in the oral cavity. For the current case, we utilized the MN technique using dermapen to enhance the absorption of topical AA (vitamin C) into pigmented gingival mucosa.

## 2. Aim of the Study

The purpose of the study was to assess the performance of the microneedling technique using dermapen with topical ascorbic acid paste on gingival depigmentation.

## 3. Case Description

**Figure 1:**
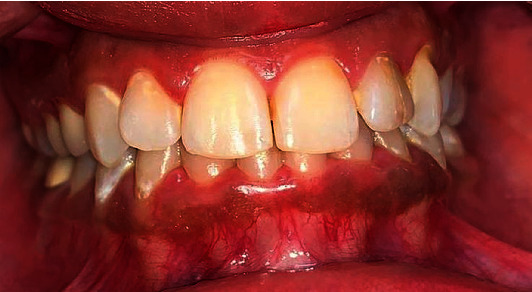
Preoperative picture of moderate gingival pigmentation.

A healthy 25-year-old female patient complained of aesthically unappealing dark colour in the lower front gum area (Figure 1). According to her detailed history, hyperpigmentation was evident from birth, which implied physiological hyperpigmentation. In addition, it was reported that the patient had never taken any medications or been diagnosed with any systemic conditions which would affect the gingival colour. Besides, her family and social histories were insignificant. According to her, she had never smoked or been exposed to second-hand smoke. Furthermore, her complaint had not been addressed in an oral setting previously.

## 4. Clinical Findings and Diagnostic Assessment

On clinical examination, no abnormalities were identified extraorally. Intraoral examination revealed a minimal accumulation of plaque and supragingival calculus, indicating an acceptable level of oral hygiene. She had an average plaque index of 0.8 and an average gingival index of 1 with no bleeding upon probing. Neither clinical attachment loss nor radiographic bone loss was determined. There was only mild marginal gingival inflammation accompanied by rolled margins and blunted interdental papillae.

A dark brown ribbon of hyperpigmentation was noticed within the gingival mucosa of the mandibular arch confined to the attached gingiva region from right to left 2nd premolars (Figure 1). The case was diagnosed as physiological moderate gingival pigmentation (pigmentation index score = 3) according to the Dummett-Gupta Oral Pigmentation Index [4] with extended ribbon-like melanin pigmentation (melanin index class 2).

## 5. Therapeutic Intervention

The patient was most concerned about achieving aesthetic results with minimally invasive, nonexpensive procedures. In light of the patient's concerns, the MN technique was suggested. The patient was provided with a detailed explanation of this off-label procedure, its instructions, and potential complications. Informed consent was established as well as permission for this off-label procedure, photos, and publication.

**Figure 2:**
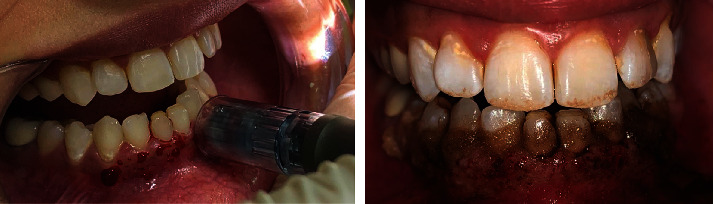
Microneedling technique using dermapen and ascorbic acid (vitamin C) application.

As a preliminary routine, supragingival scaling was performed and oral hygiene instructions were provided by the dental hygienist. We subsequently scheduled the procedure and performed it aseptically. Local anaesthesia 2% lignocaine (1 : 80,000 adrenaline) was administered by infiltration technique in the mandibular region. To microneedle the gingival mucosa, we used a dermapen device model M8 with 24 microneedles arranged in rows, which was adjusted with 1.5 mm depth at the 6^th^ mode speed of 700 cycles/min. The dermapen was used in intermittent motion on the sextant gingival area for 30-40 seconds/tooth. When bleeding pinpoints were observed on all areas of pigmented gingiva, the gingival mucosa was irrigated with a saline solution and sterile gauze was applied to dry the area. Then, topical AA powder (1000 mg/ml) was mixed with saline in a small glass dish forming a paste. The mixed slurry paste was applied to the gingival mucosa using for 10 minutes as shown in Figure 2. The treated area was left without dressing. Our patient was instructed to refrain from drinking acidic or hot beverages for 24 hours and to not brush her lower teeth for one day to avoid any mechanical trauma to the gingiva. Following the procedure, neither mouthwashes nor medications were prescribed for her. The photos were taken before, during, and at follow-up appointments until 6 months.

## 6. Clinical Follow-Up and Outcomes

**Figure 3:**
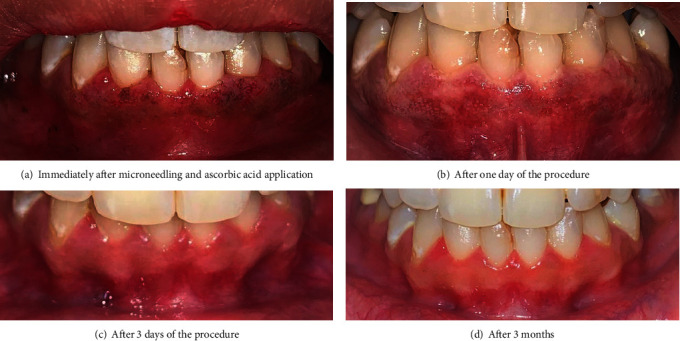
(a–d) Postoperative pictures of gingival depigmentation.

**Figure 4:**
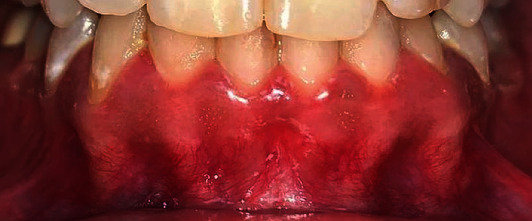
Recurrence of gingival pigmentation started after 6 months.

One day after the procedure, the attached gingiva exhibited an increase in volume and an alteration in texture as well as the appearance of white and red-coloured areas, indicating tissue inflammation (Figure 3(a)). Neither pain nor tenderness was reported, and only discomfort sensation was experienced. On the third day, all of these findings diminished resulting in the pink appearance of the gingiva (Figure 3(b)). The same procedure was performed after 2 weeks, resulting in same the postoperative tissue inflammation which diminished on the 3^rd^ day of the second application. Clinical outcomes showed complete disappearance of gingival pigmentation. Generally, after the first and second application, healing was normal and satisfactory providing excellent aesthetic results where the pigmentation index score decreased to zero with reduction of melanin index (class 0) as presented in Figures 3(c) and 3(d). After the 6^th^ month of the follow-up period, repigmentation of the gingiva was detected showing light brown solitary pigmented areas as depicted in Figure 4.

## 7. Discussion

Although the GP does not exhibit any medical concerns, patients frequently complain of “black gums” seeking cosmetic solutions. In this case presentation, the patient was diagnosed with physiological pigmentation based on the negative history of any pathological cause, since the physiologic pigmentation is probably genetic in nature.

GD is considered a periodontal plastic procedure whereby the gingival hyperpigmentation is removed or reduced through various techniques such as scalpel scraping, bur abrasion, free gingival graft, cryosurgery, electrosurgery, and laser [6]. In the present case, we achieved GD by a novel technique using microneedling and topical AA. It is a minimally invasive, well-tolerated, safe, not expensive, and less time-consuming procedure.

To our knowledge, this is the first study to introduce NM with AA in gingival depigmentation. Nevertheless, there was a study done by Ozsagir et al. [15] who compared the use of i-PRF alone or in combination with microneedling for gingival augmentation. They concluded that after 6 months, the group using i-PRF combined with microneedling showed a statistically significant increase in gingival thickness compared to the group using only i-PRF.

Although, the FDA has legally authorized microneedling devices to improve the appearance of facial acne scars, facial wrinkles, and abdominal scars in patients aged 22 years or older, these devices are still considered as off-label procedures for oral use. However, there are various types of MN devices including dermaroller, dermastamp, and dermapen. Unlike other MN devices, dermapen is the only device that can be applied intraorally due to its small size, changeable head. This increases the accessibility inside the mouth and makes it easy to be used for a larger number of patients. This device is a wireless electric device with automated features that allow the operator to modify the speeds, pressures, and depth of penetration, thus reducing the probability of operator-related side effects.

In the present study, the patient agreed and signed explicit written consent for this off-label procedure being used as a part of research. During the procedure, it was necessary to generate precise bleeding pinpoints by MN to achieve optimal results [16]. These microholes allow the therapeutic medication to penetrate easily into the connective tissues, stimulating new collagen production and affecting melanocytes [8].

Then, we used topical AA as a therapeutic medication that was reported to be effective in reducing melanogenesis directly and thus promoting depigmentation, because melanin acts as a reservoir for reactive oxygen species (ROS), Cu, and calcium within the cells. Following AA entry into the target tissue, it binds to melanin causing a deficiency of ROS, Cu, and Ca, resulting in the reduction in melanin production [17]. As to say that AA affects melanocyte function rather than the number, as opposed to other approaches relying on the destruction of melanocytes.

Our outcomes revealed excellent aesthetic results, and these were in agreement with Yussif et al. [18] who used intraepithelial injections of 1-1.5 ml AA (200-300 mg) in the gingival mucosa and reported that its direct delivery reduced pigmentation incidence scores and area of pigmentation. As well as comparing injectable AA to conventional scalpel depigmentation by Yussif et al. [19] in 2019 who concluded that injecting AA into pigmented cells presented comparable results to conventional surgery.

Furthermore, our findings were consistent with the results of Shimada et al. [20] who applied topical vitamin C gel to the gingiva and concluded that AA inhibited melanin pigmentation. However, a study made by El-Mofty et al. [21] stated that intramucosal AA injections were better and more efficacious than topical AA gels for GD.

Following the first day of the procedure, the gingiva showed an increase in volume and a change in texture with white and red-coloured areas representing gingival inflammation, oedema, and irritation. These results were corresponding with numerous clinical studies in dermatology which revealed that the MN technique can cause postoperative transit oedema and erythema that resolves usually after 1-3 days [22–25].

In contrast to any other depigmentation procedure, the patient was able to see these excellent aesthetic results after 3 days (Figure 3). However, healing after scalpel depigmentation takes 7-10 days, and other procedures take more than 2 weeks to heal [26, 27]. In addition, a dressing is not necessary after the procedure since microholes are created without perfused bleeding and heal quickly, unlike scalpel depigmentation where the gingiva should be covered with dressing as it leaves denuded connective tissue and heals by secondary intention [5]. Moreover, a free gingival graft can be used for GD, but it involves a second surgical site (donor site) and it has unaesthetic colour matching [28].

Compared to other minimally invasive techniques, our technique poses less risk of complications and is less expensive. However, lasers are considered easy, fast, and efficient modalities that have hemostasis and decontamination effects, but they are expensive and tissues heal within 1-2 weeks [29, 30]. Additionally, electrosurgery has been reported to cause heat accumulation and undesired tissue destruction, and it takes 5-14 days for complete healing [31]. While cryosurgery may cause postoperative swelling and increase soft tissue destruction as well as difficulty in controlling the depth and freezing duration, also it takes 3-4 weeks for complete keratinization [26].

Pigment recurrence is described as a spontaneous and recurrent process that occurs within 24 hours up to 8 years following the depigmentation procedure which poses a challenge to a dentist [28]. Methods of treatment, number of recall periods, genetic and ethnic factors, tobacco consumption, and hormonal factors influence the gingival repigmentation [32]. It has been hypothesized that the rate of melanin formation is higher in darker individuals [33]. Also, gingival pigmentation is more in the anterior gingiva than posterior ones which have been attributed to sunlight exposure [34]. Many authors [35–37] who used surgical technique showed early recurrence of pigmentation after 15-56 days of follow-up periods. However, the majority of the available literature has shown a lower recurrence rate for cryosurgery and lasers [38]. Nakamura et al. [39] described repigmentation after 24 months after laser depigmentation. Additionally, previous studies [40–43] have reported gingival pigmentation recurrence after 12-48 months following cryosurgery depigmentation.

In our case report, repigmentation was observed after 6 months (Figure 4). This might be due to the proliferation of melanocytes and their migration to the depigmented area [27]. These recurrence findings vary from those of Sheel et al. [44] who used scalpel depigmentation along with the monthly local application of AA, which gave positive aesthetic results, but recurrence was observed after 9 months. This may be related to the amount of AA that gingival tissues are exposed to.

However, our procedure is well tolerated by patients and can be repeated from time to time for more aesthetic results.

## 8. Conclusions

Clinical experience, affordability, and personal preferences should be considered when selecting a technique for treating gingival hyperpigmentation. Our novel technique of using dermapen and topical ascorbic acid in the treatment of gingival pigmentation showed promising results, which gives new perspectives for its application. Although our technique is a simple and safe GD method, it should be performed cautiously; otherwise, tissue destruction and gingival recession may result. Our results were limited to this particular case. Therefore, the present findings should be confirmed in future randomized controlled clinical trials, including histological analysis.
